# The *De Novo* Genome Assembly of *Olea europaea* subsp. *cuspidate*, a Widely Distributed Olive Close Relative

**DOI:** 10.3389/fgene.2022.868540

**Published:** 2022-08-25

**Authors:** Tao Wu, Ting Ma, Tian Xu, Li Pan, Yanli Zhang, Yongjie Li, Delu Ning

**Affiliations:** Institute of Economic Forest, Yunnan Academy of Forestry and Grassland, Kunming, China

**Keywords:** *Olea europaea* subsp*. cuspidate*, olive subspecies, wild olive, genome, Hi-C

## Abstract

The olive complex, comprising six subspecies, is a valuable plant for global trade, human health, and food safety. However, only one subspecies (*Olea europaea* subsp. *europaea*, OE) and its wild relative (*Olea europaea* subsp*. europaea* var*. sylvestris*, OS) have genomic references, hindering our understanding of the evolution of this species. Using a hybrid approach by incorporating Illumina, MGI, Nanopore, and Hi-C technologies, we obtained a 1.20-Gb genome assembly for the olive subspecies, *Olea europaea* subsp. *cuspidate* (OC), with contig and scaffold N50 values of 5.33 and 50.46 Mb, respectively. A total of 43,511 protein-coding genes were predicted from the genome. Interestingly, we observed a large region (37.5 Mb) of “gene-desert” also called “LTR-hotspot” on chromosome 17. The gene origination analyses revealed a substantial outburst (19.5%) of gene transposition events in the common ancestor of olive subspecies, suggesting the importance of olive speciation in shaping the new gene evolution of OC subspecies. The divergence time between OC and the last common ancestor of OE and OS was estimated to be 4.39 Mya (95% CI: 2.58–6.23 Mya). The pathways of positively selected genes of OC are related to the metabolism of cofactors and vitamins, indicating the potential medical and economic values of OC for further research and utilization. In summary, we constructed the *de novo* genome assembly and protein-coding gene pool for *Olea europaea* subsp. *cuspidate* (OC) in this study, which may facilitate breeding applications of improved olive varieties from this widely distributed olive close relative.

## Introduction

As “the queen of vegetable oils” and “a symbol of peace,” *Olea europaea* subsp. *europaea* (OE) is one of the most widespread and socioeconomically important oil crops in the Mediterranean Basin. It is well-acknowledged that olive domestication is one of the most important events in human agricultural civilization. This event was initiated in the Near East around 4,000–6,000 years ago, and now olive trees have been planted in more than 40 countries due to its distinguished nutritional value (Kostelenos et al., 2017). Apart from its agricultural and economic importance, olive oil also has great medical importance due to its high-value health compounds, including monounsaturated free fatty acids, squalene, phytosterols, and phenols, which may exert favorable effects on inflammation, free radicals, gut microbiota, and carcinogenesis (Borzì et al., 2019).

Although genome-wide features of this species have been investigated extensively (Cruz et al., 2016; Rao et al., 2021), *O. europaea* is not a singular and isolated species. OE is one member of the olive compound species, a well-known species complex with a total of six subspecies members. This evolutionary complexity renders the subspecies nearly comparable in scientific relevance due to their close and complicated relationship. The six natural subspecies distribute over a wide range of the Old World and comprise OE (the cultivated olive), which was genetically domesticated from “wild olive” (*Olea europaea* subsp. *europaea* var*. sylvestris*, OS), and the other five wild relatives (subsp. *cuspidate*, OC; subsp. *laperrinei*; subsp. *maroccana*; subsp. *cerasiformis,* and subsp. *guanchica*) (Green, 2002; Sebastiani and Busconi, 2017). The five wild relatives of OE thrive in Africa, Asia, Europe, and the islands of the Indian Ocean (Mauritius and Madagascar) (Besnard et al., 2013).

Wild olive relatives also have multiple economically important and promising properties, including resistant and strong growth characteristics. Their intersubspecific hybrid with cultivated olive can amplify the genetic basis of the existing olive germplasm resources. OC has many common names, such as African olive, Indian olive, brown olive, and wild olive, probably due to its wide distribution in China, Iran, India, and at higher elevations in North, East, and South Africa (Green, 2002) and its close relationship with OE. Natural hybridization does exist between OE/OS and OC (Hannachi et al., 2008). Experimental hybrids between a domesticated olive variety and a wild relative of the same genus or subspecies were also reported in several studies (Besnard et al., 2001; Ma et al., 2014; Cáceres et al., 2015; Niu et al., 2020; Li et al., 2021). OC is frequently used as a graft rootstock for olive to provide vigor and possible resistance against olive fungal diseases. Grafting experiments in China showed that the survival rate of grafted seedlings was high, but the grafted plants were prone to “little feet,” an appearance of a big top and a small bottom at the association interface because of the slow growth of OC (rootstock) and the rapid growth of OE (scion) (Shi et al., 1991).

In recent years, the Yunnan Academy of Forestry and Grassland has successfully bred a few olive varieties by crossbreeding of *O. europaea* subsp. *europaea* cv. Frantoio as the female parent and OC as the male parent. One of these hybrid varieties, named Yunza 3 or Jinyefoxilan, characterized by a lepidote trichome under the leaf blade, was registered as a new variety of horticultural plants in Yunnan Province, China (Ma et al., 2014). The fruit of Yunza 3 is oval, the average weight of a single fruit is 1.50 g, the pulp rate is 68.90%, and the oil content of the whole fresh fruit is 16.00%. Due to its strong adaptability and high vigor in southwest China (Yunnan province), this hybrid variety is extensively used as rootstock. The survival rate of grafted olive is high, without the “little feet” phenomenon (Ma et al., 2014). This breeding achievement strongly demonstrates the great potential of OC to improve the agroeconomic traits of olive.

To cultivate and improve new olive varieties based on intersubspecific crossing, it is necessary to further understand the genomic information of more wild subspecies. By November 2021, the whole genomes of three olive varieties from OE [cv. Leccino (Barghini et al., 2014), cv. Farga (Cruz et al., 2016), cv. Arbequina (Rao et al., 2021)], and a wild olive tree from OS (called oleaster, *Olea europaea* subsp. *europaea* var*. sylvestris*) (Unver et al., 2017) have been sequenced. The genome information of OC is not available, except for chloroplast genome data (Besnard et al., 2011). Despite the agricultural importance, there is still no high-quality genome reference for OC (subsp*. cuspidata*). There is no doubt that the reference genome has fundamental importance in aiding the target-gene sequencing and short-read mapping and in molecular breeding, population diversity, and genotype–phenotype association study. The lack of this basic data strongly hinders our understanding of genomic evolution, diversity, oil biosynthesis, and local adaptation of this important plant complex. Here, we studied the genome of subsp. *cuspidata* by incorporating Illumina, MGI, Nanopore, and Hi-C technology, which would provide insights on the adaptive evolution, molecular breeding, genomic novelty, and phylogenetic relationship of the olive complex.

## Materials and Methods

### Sampling, Sequencing, *De Novo* Assembling, and Annotation

The taxonomy of the investigated OC sample was identified by Dr. Yong-Kang Sima, a professional taxonomist from the Yunnan Academy of Forestry and Grassland. This sample is now deposited in Kunming Arboretum, Yunnan province of China (voucher specimen Wu20056, N 25°9′13″, E 102°45′9″). The standard preparation procedures before sequencing, including DNA and RNA extraction and Hi-C library construction, were based on the requirements of specific sequencers. In total, five tissues, namely, leaves, roots, twigs, bark, and fruits were used for RNA-seq in Illumina platform. For DNA-seq, 65.68 Gb short-reads (300 bp PE) and 96.5 Gb Nanopore long-reads were obtained from the DNBSEQ-T7 and PromethION platform, respectively. The raw reads were filtered using the fastp preprocessor (Chen S. et al., 2018). To achieve chromosome-level assembly, we further generated 129.21 Gb data of the paired-end Hi-C reads (150 bp) from the DNBSEQ-T7 platform (MGI). We conducted the karyotyping of OC to determine the number of chromosomes using rooted cuttings, which have active meristems of mitosis suitable for detecting clear chromosomes. The root tips were treated with nitrous oxide to obtain sufficient cells at mitosis metaphase for staining with DAPI and telomere repetitive sequences (TTTAGGG) 6.

A genome survey was conducted using GenomeScope (Vurture et al., 2017) for heterozygosity and repeat content. The genome size was estimated with the mean values of gce 1.0.2 with *k-mer* 17, 19, and 21 (Liu et al., 2013). The basecalling output from the PromethION platform was treated using Guppy (Wick et al., 2019). Only the reads with mean quality scores >7 were retained and further corrected using NextDenovo software with parameters “reads_cutoff:2k, seed_cutoff:18k” (https://github.com/Nextomics/NextDenovo) (Wang et al., 2019). The assembling processes include the correction module using NextCorrect and the assemble module using NextGraph, with default parameters. Subsequently, Nextpolish software was used to polish the genome with short-reads four times and long-reads three times (sgs_options = -max_depth 100) (Hu et al., 2020). The paired-end Hi-C reads were filtered by fastp to remove the adapter and low-quality reads (Phred Score >15, and 5 > number of Ns in the reads) (Chen S. et al., 2018). The obtained assembly was further corrected with 3d-DNA five times and manually tuned with Juicebox Assembly Tools v1.9.8 (Dudchenko et al., 2018). During scaffolding, to facilitate contig ordering and revise the misjoin, we mapped the OC draft genome to OE assembly using minimap2 with parameter “-xasm10” (Li, 2018) and visualized the major structure variations with dotPlotly (https://github.com/tpoorten/dotPlotly). Subsequently, the pseudo-structural variations caused by misassembling were manually corrected by examining the HI-C matrix with Juicebox following the official manual (https://aidenlab.org/assembly/manual_180322.pdf). The genome assessments were conducted by using LTR_retriever (Ou et al., 2018), mapping rate of short-read data by BWA (Li and Durbin, 2009), and N50 values with QUAST (Gurevich et al., 2013), with default parameters.

The RepeatMasker v2.0.3 was used for repeat annotation following the manual-recommended parameters (Tarailo-Graovac and Chen, 2009). To aid gene annotation, a total of ∼25 Gb RNA-sequencing (RNA-seq) clean pair-ended reads from five tissues, namely, leaves, roots, twigs, barks, and fruits were generated using Illumina HiSeq platform. All libraries were *de novo* assembled separately and subsequently merged using the TransABySS v2.0.1 manual pipeline (Robertson et al., 2010). The protein-coding and non-coding gene structural annotation was conducted using the MAKER2 pipeline (Cantarel et al., 2008) by incorporating transcriptome mapping, *de novo* gene predictions, and homology predictions with OS proteins from the NCBI (GCF_002742605.1). The majorly used softwares from MAKER2 pipeline include blast + tools (Camacho et al., 2009), exonerate v2.2.0 (Keller et al., 2011), hmm-E and GeneMark-ES (Borodovsky and Lomsadze, 2011), and augustus (Stanke and Morgenstern, 2005).

The high-throughput sequencing data files are available at the GenBank database (https://www.ncbi.nlm.nih.gov/), with SRA accession numbers: SRR17299471 and SRR17299472. The associated BioProject and BioSample numbers are PRJNA785068 and SAMN23526758, respectively. The genome assembly of OC is available under NCBI accession number JAKWBP000000000.

### Gene Family and Species Evolution

For species evolution, we organized “dataset A″ to address the questions related to phylogeny and divergence time. The dataset A covers three subspecies of olive (OC, OS, and OE), in addition to the other five species of eudicots without gene annotations. Five species, namely, *Jasminum sambac, Forsythia suspensa*, *Fraxinus pennsylvanica*, *Fraxinus excelsior*, and *Osmanthus fragrans* with reference genomes but without gene annotations were retrieved from the NCBI (Supplementary Table S1A). *Arabidopsis thaliana* was further added as an outgroup species. To facilitate species phylogeny analysis, we used a “proxy” approach based on dataset B, which involves 10 species with available gene annotations from the NCBI (Supplementary Table S1B). In detail, these species/subspecies include *Arabidopsis thaliana*, *Arachis hypogaea*, *Elaeis guineensis*, *Glycine max*, *Helianthus annuu*s, *Juglans sigillata*, *Ricinus communis*, *Sesamum indicum*, *Olea europaea* subsp. *europaea* var*. sylvestris*, and *Olea europaea* subsp. *europae* cv “Arbequina” (Supplementary Table S1B).

In simple terms, the strategy of the “proxy” approach is that the “one-to-one” single-copy orthologous genes were identified from dataset B and then mapped to dataset A to re-analyze orthologous gene groups. In detail, based on the “one-to-one” orthologous genes obtained from dataset B with OrthoFinder v2.5.4 (Li et al., 2003), we locally annotated the corresponding homologous genes for dataset A using BRAKER2 with only homology prediction (Brůna et al., 2021). Then, these homologous genes were fed into OrthoFinder v2.5.4 again to obtain “one-to-one” orthologous single-copy genes for dataset A (Supplementary Table S2).

In detail, the orthologous genes, phylogeny, and divergence time were analyzed as follows. The OrthoFinder v2.5.4 with default parameters was used for gene family and orthologous gene identification (Li et al., 2003). Only the longest transcript was used for protein sequence comparison with BLAST tools (Altschul et al., 1997). We estimated the evolutionary topology with FastTree-2 (Price et al., 2010), an approximately maximum-likelihood (ML) method, using the combined sequences of “one-to-one” single-copy gene families, with bootstrap replicates set to 1,000. MCMCTREE in PAML v4.8a was used to estimate the divergence time of these species (Yang, 2007). The divergence calibration was based on the divergence time between “*Osmanthus fragrans*” and “*Olea europaea*” (7-45Mya) from the time-tree database (http://www.timetree.org). The sequence alignment and filtering were based on MAFFT v7.49 (Katoh and Standley, 2013) and Glocks (parameter: b5 = h) (Castresana, 2000).

For gene family evolution, we only analyzed dataset B (Supplementary Table S3), which has available gene annotations from the NCBI. The CAFE v4.2.1 (Computational Analysis of gene Family Evolution) package (De Bie et al., 2006) was used to analyze gene family expansion and contraction with a significant level of *p*-value < 0.01 across ancestral nodes, leading to olive species.

#### Fast Evolution and Positive Selection Analysis

Identifying genes under positive selection is a common way to detect genes with novel functions and molecular adaptation, which has been successfully applied in both plants and animals (Yang and Dos Reis, 2010; Zhang et al., 2011; Chen J. et al., 2018). In this study, the branch model and branch-site model in PAML packages (v4.8a) were used to detect fast evolution and positively selected genes, based on dataset B with available gene annotations. The branch model was analyzed by comparing the “free-ratio model” with the “one-ratio model” and choosing only the significant genes and those evolving fastest in OC. The subsequent genes were identified by comparing Model A (assuming the focal branch under positive selection indicated by Ka/Ks > 1) with the null model (Ka/Ks of any site was ≤1). The statistical significance of the likelihood ratio test (LRT) was determined with “chi2” function in PAML. The positive selected sites were further determined using the Bayesian method (BEB, Bayes empirical method) with a probability value of over 0.95.

### Whole-Genome Duplication and Transposed Gene Duplications

Whole-genome duplication (WGD) analysis was conducted by the 4DTv method (four-fold synonymous third-codon transversion) and Ka/Ks estimation in MCScanX with default parameters (Wang et al., 2012). The gene duplication event dating was determined using MCScanX-transposed (Wang et al., 2013). Specifically, the gene duplication types were categorized into tandem duplication, proximal duplication, segmental duplication, and transposed gene duplication. The oldest branch of the synteny block was used as a proxy for the gene ages of transposed genes. The retrogenes, or the RNA-based gene duplications, were identified using a method similar to that used by Betrán et al. (Betrán et al., 2002), with the BLASTP parameters including identity value >60%, length mapping coverage >80%, and an E-value < 0.000001.

### Structural Variation Identification

We first tried the SyRI and the “assembly-to-assembly” approach for SV identification (Chen et al.; Goel et al., 2019). However, these approaches are better for references at the population level or with higher DNA identity. We further conducted the SV identification based on comparing OC long reads to OS and OE references with a dual-mode alignment strategy. In detail, the reads were mapped to a reference with two commonly used mappers, Minimap2 and NGMLR, which are integrated in a software named Vulcan (Fu et al., 2021). Minimap2 is a highly fast long-read mapper, implementing a time-efficient alignment approach involving a two-piece affine gap model and a faster chaining process (Li, 2018). NGMLR is designed to make use of a convex scoring matrix to better distinguish the read error from the SV signal (Sedlazeck et al., 2018). For SV calling, we utilized Sniffles (version 2.0.3) and filtered out imprecise and low reads supporting SVs (<3) (Sedlazeck et al., 2018).

## Results and Discussion

### Genome Assembly of *Olea europaea* subsp*. Cuspidata*

Before performing *de novo* genome assembly, we estimated the genomic featuring parameters including genome size, heterozygosity, and repeat content to roughly assess the complexity of the *O. europaea* subsp*. cuspidata* genome with *k-mer* analysis (Chor et al., 2009; Liu et al., 2013; Vurture et al., 2017), which is the most frequently used method for genomic survey. Compared to the previously reported OE genome (Rao et al., 2021), OC has a higher level of heterozygosity (2.28% vs. 1.09%), a comparable level of repeat content (54.5% vs. 56.18%), and a slightly smaller genome size (1.2G vs. 1.3G).

**FIGURE 1:**
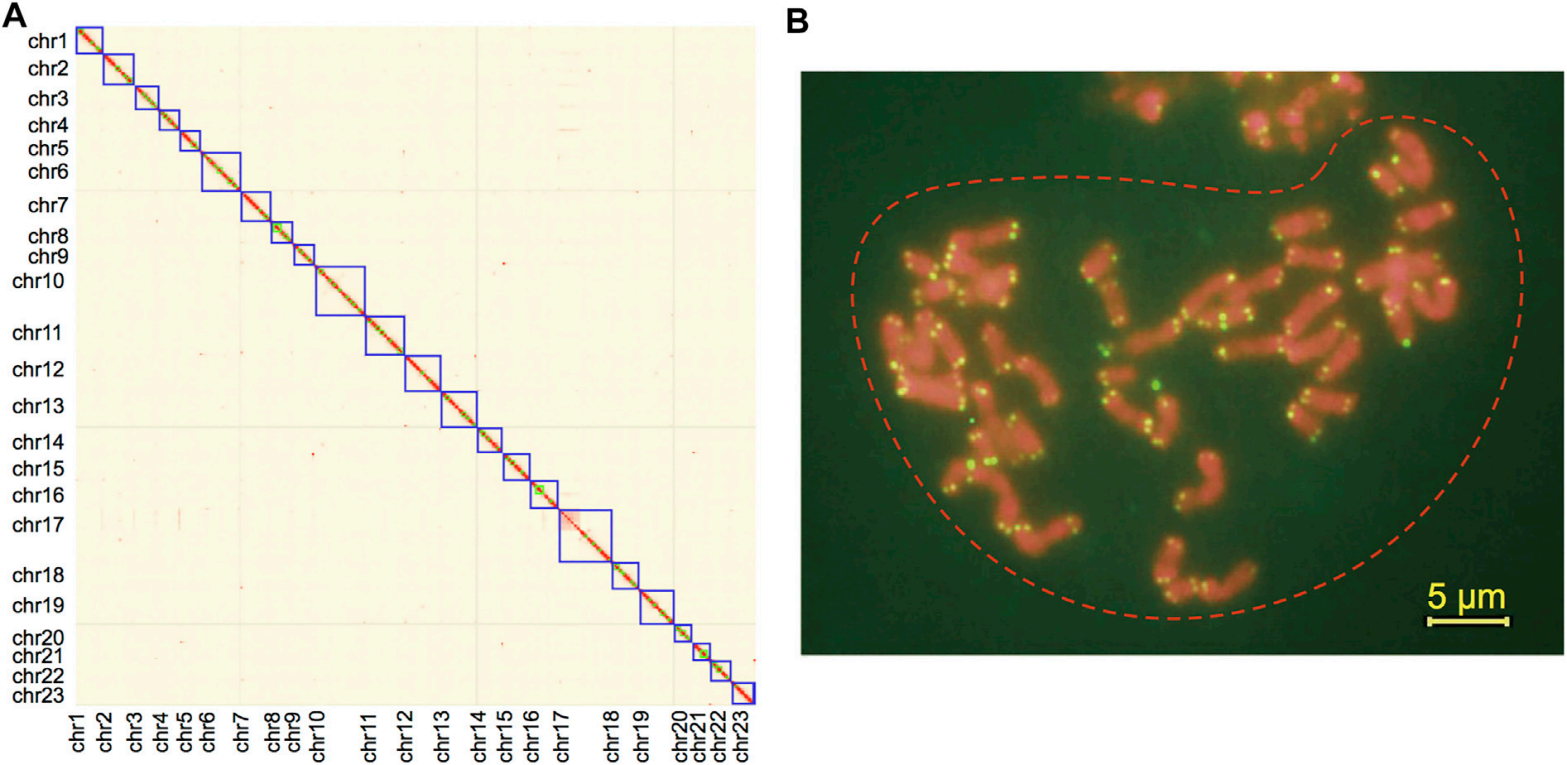
**(A)** Intensity signal heatmap of the Hi-C chromosome for *Olea europaea* subsp. *cuspidata* (OC). **(B)** Karyotype by telomere staining with repetitive sequences (TTTAGGG)6. Note: the yellow bar indicates 5 μm.

In total, 65.68-Gb MGI DNA-seq short reads (300 bp PE, 54.7×), 129.21-Gb Hi-C paired-end reads (107.7×), and 96.5-Gb Nanopore long-reads (80.4×) were obtained following data filtering. The draft contigs were constructed with short reads and Nanopore long reads, followed by semi-automatic scaffolding with 3D-DNA (Dudchenko et al., 2018). After manually revising the orders and orientations of super-scaffolds with Hi-C interaction signals, we achieved an anchor rate of 87.95% to place the initial contigs to scaffolds. We observed a clear aggregation of 23 super-scaffolds, which are also OC chromosomes, with the lengths from 28.38 to 87.93 Mb (Supplementary Table S4). All other scaffolds or contigs are shorter than 0.8 Mb and have no clear signals of interaction with any chromosome (Figure 1A). We further validated the total chromosome number of 23 in OC by using the karyotyping of DAPI staining (Supplementary Figure S1) and telomere staining with repetitive sequences (TTTAGGG) 6 (Figure 1B).

The genome size of the final OC reference was estimated to be 1.20 Gb. The longest scaffold and contig are 87.93 and 17.29 Mb, respectively. The lengths of the contig and scaffold at 50% of total genome length (N50) are 5.33 and 50.46 Mb, respectively (Table 1), which are greater than those of the previously published wild olive tree oleaster (contig N50, 25.49 Kb; scaffold N50, 228.62 Kb) (Unver et al., 2017) and the cultivated *Olea europaea* genome (contig N50, 4.67 Mb; scaffold N50 42.60 Mb) (Rao et al., 2021). We mapped the original clean short and long reads to the novel *de novo* OC genome assembly which was used as a reference. The mapping rate of MGI short-read data against the OC reference is 99.91%, which is almost the same as that of Nanopore long reads (99.38%). In addition, the LAI score (12.95) indicates the high quality of the OC genome that has reached the reference level, based on previous assessment of multiple species (Ou et al., 2018).

**TABLE 1:** Summary of the *de novo* genome assembly of OC and the comparison with two related species, *Olea europaea* subsp. *europaea* var. *sylvestris* (OS) and *Olea europaea* subsp. *europae* cv “Arbequina” (OE).

Assembly	OC	OS	OE
No. of sequences (≥ 50,000 bp)	187	2,104	849
No. Total length (≥ 50,000 bp)	1,183,913,677	985,700,118	1,098,745,707
No. of sequences	1,078	41,219	962
Largest sequence (bp)	87,931,667	46,026,434	68,066,766
Total length (bp)	1,196,933,720	1,141,142,775	1,102,969,454
GC (%)	35.36	35.4	34.33
N50 (bp)	50,460,234	12,567,911	42,601,851
N75 (bp)	41,133,639	174,775	35,395,138

### Annotation of the *O. europaea* subsp. *Cuspidata De Novo* Genome Assembly

To evaluate the continuity of both assembly and protein-coding genes, we conducted BUSCO analysis to assess the completeness and redundancy of the OC assembly and proteins based on the fractions of conserved genes (Manni et al., 2021). BUSCO assessment revealed that 94.1% of 1,440 plant conserved genes are complete in OC assembly, similar to the level of the OE assembly reported previously (Rao et al., 2021) and much higher than that of OS. Similar patterns were found for assembly and protein completeness (Table 2), suggesting high level of integrity and completeness of the OC genome. The repeat annotation based on RepeatMasker revealed that repeats, including DNA elements, LINE, SINE, LTR, satellite, simple repeats, and unknown elements, account for 74.22% of genome sequences (Table 3), which is higher than the estimation based on the *k-mer* survey. The top three abundant repeat elements are LTR, DNA elements, and LINE, accounting for 62.76%, 11.03%, and 2.48%, respectively (Table 3).

**TABLE 2:** BUSCO assessment of genome and gene continuity.

	Assembly proteins	Percentage (%)	Annotation proteins	Percentage (%)
Complete BUSCOs	1,356	94.1	1,393	96.7
Complete Single-Copy BUSCOs	1,036	71.9	997	69.2
Complete Duplicated BUSCOs	320	22.2	396	27.5
Fragmented BUSCOs	20	1.4	25	1.7
Missing BUSCOs	64	4.5	22	1.6
Total BUSCO groups searched	1,440	100	1,440	100

**TABLE 3:** Annotation summary statistics for repeats of the OC reference genome.

Type	Repbase TEs (%)	TE proteins (%)	De novo (%)	Combined TEs (bp)	Combined TEs (%)
DNA elements	1.73	0.74	9.76	157,780,557	11.03
LINE	0.37	0.24	2.19	35,494,175	2.48
SINE	0	0	0.06	922,395	0.06
LTR	16.29	12.32	61.52	897,920,339	62.76
Satellite	0.16	0	0.29	6,373,488	0.45
Simple repeat	0	0	0.02	348,505	0.02
Unknown	0.01	0	3.77	32,645	3.79
Total	18.37	13.3	72.14	54,150,429	74.22

**FIGURE 2:**
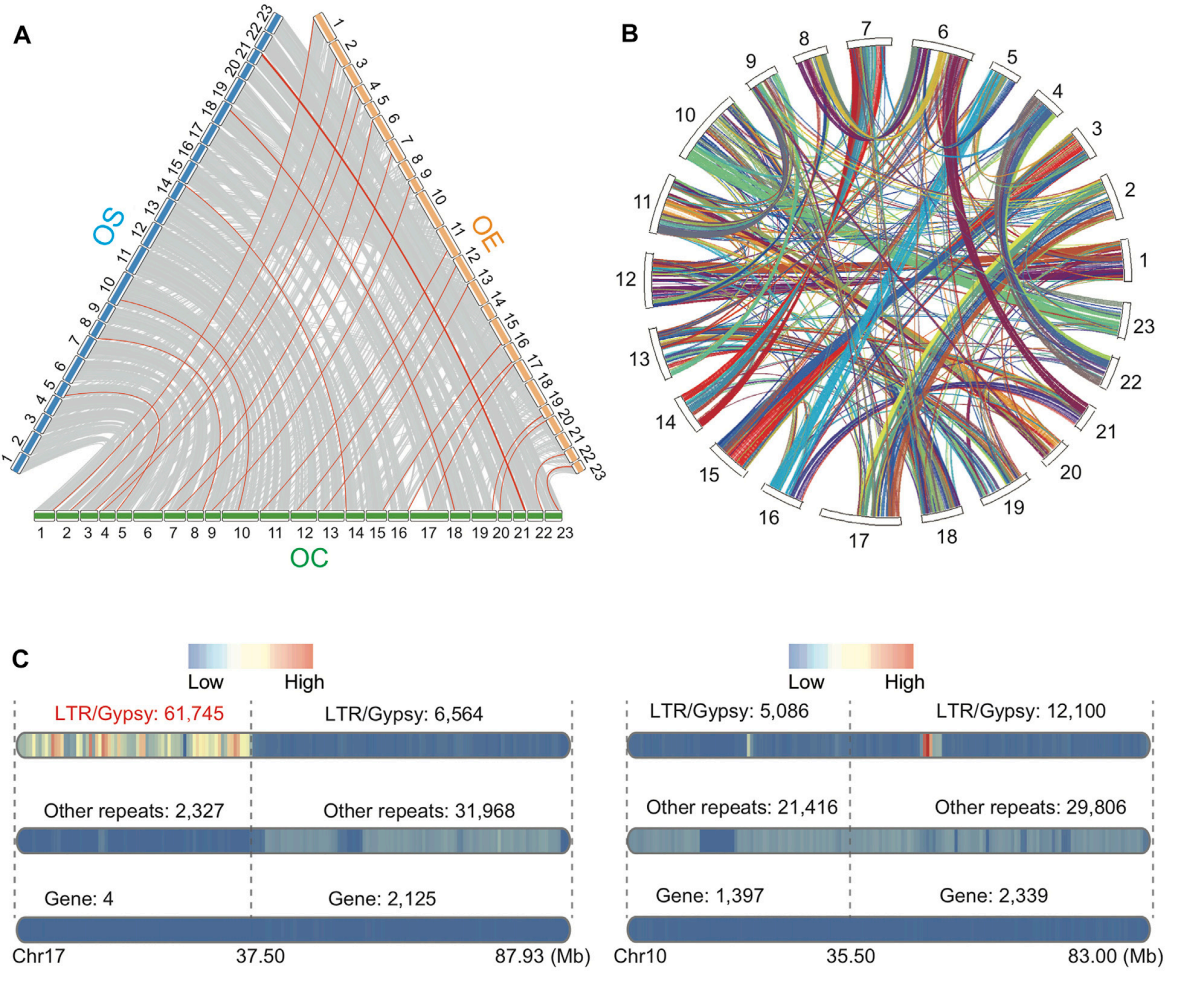
**(A)** Whole-genome alignment among OC, OS, and OE based on Minimap2 (>500 Kb). Note: the red lines show the highest DNA identity values per base pair between OC and OE or between OC and OS. **(B)** Collinear blocks within the OC genome based on BLASTP and MCScanX (evalue 1e-3). **(C)** Visualization of genes and repeats for chromosome 17 (left) with a focus on the 37.5-Mb region and chromosome 10 as a control (right) due to comparable chromosome size. The numbers of LTR/Gypsy, other repeats, and genes are shown above each bar.

To understand inter- and intra-assembly synteny, we conducted a whole-genome alignment between OC and OE and between OC and OS based on Minimap2 (Li, 2018) (Figure 2A). We also conducted self-alignment using MCScanX software with collinear genes of OC (Wang et al., 2012) (Figure 2B). The cross-assembly comparison revealed that OC has the highest alignment identity rates to OS rather than OE, suggesting closer distance from OC to wild olive (OS) than from OC to domestic olive (OE) (Figure 2A). Unexpectedly, based on these two alignments, we found a “gene-desert” region on chromosome 17 of OC (0–37.5 Mb, Figure 2C). Only four genes, including phytochrome B–like gene, transposable element gene, arginine methyltransferase–interacting related gene, and zinc finger BED domain–containing related gene, are found within this region. BLASTP search against the database of RefSeq non-redundant proteins revealed that these genes are genetically nearest to OS, consistent with the overall pattern of the other chromosomes. Among the four genes, the phytochrome B–like gene and the arginine methyltransferase–interacting related gene are particularly interesting due to their known roles in light-controlled chromatin compaction and methylation regulation (Tessadori et al., 2009; Cho et al., 2012; Zhang et al., 2019). In addition, we uncovered that this “gene-desert” region is also the “LTR-hotspot”, with the highest density of retrotransposon LTR/Gypsy (61,745/63,462, 97.29%) among all types of repeats (Figure 2C). Interestingly, within chromosome 17, 90.39% of LTR/Gypsy repeats reside in the 37.5-Mb region (61,745 out of 68,309), suggesting significant local enrichment (χ^2^ test, *p* < 0.00001). This region covers 2.98 to 16.40 times higher number of LTR/Gypsy (61,745) than other complete chromosomes, which range from 3,766 in chromosome 21 to 20,731 in chromosome 6. This finding may pave the way for future study on the olive region of “gene-desert” but “LTR-hotspot”.

### Whole-Genome Duplication

**FIGURE 3:**
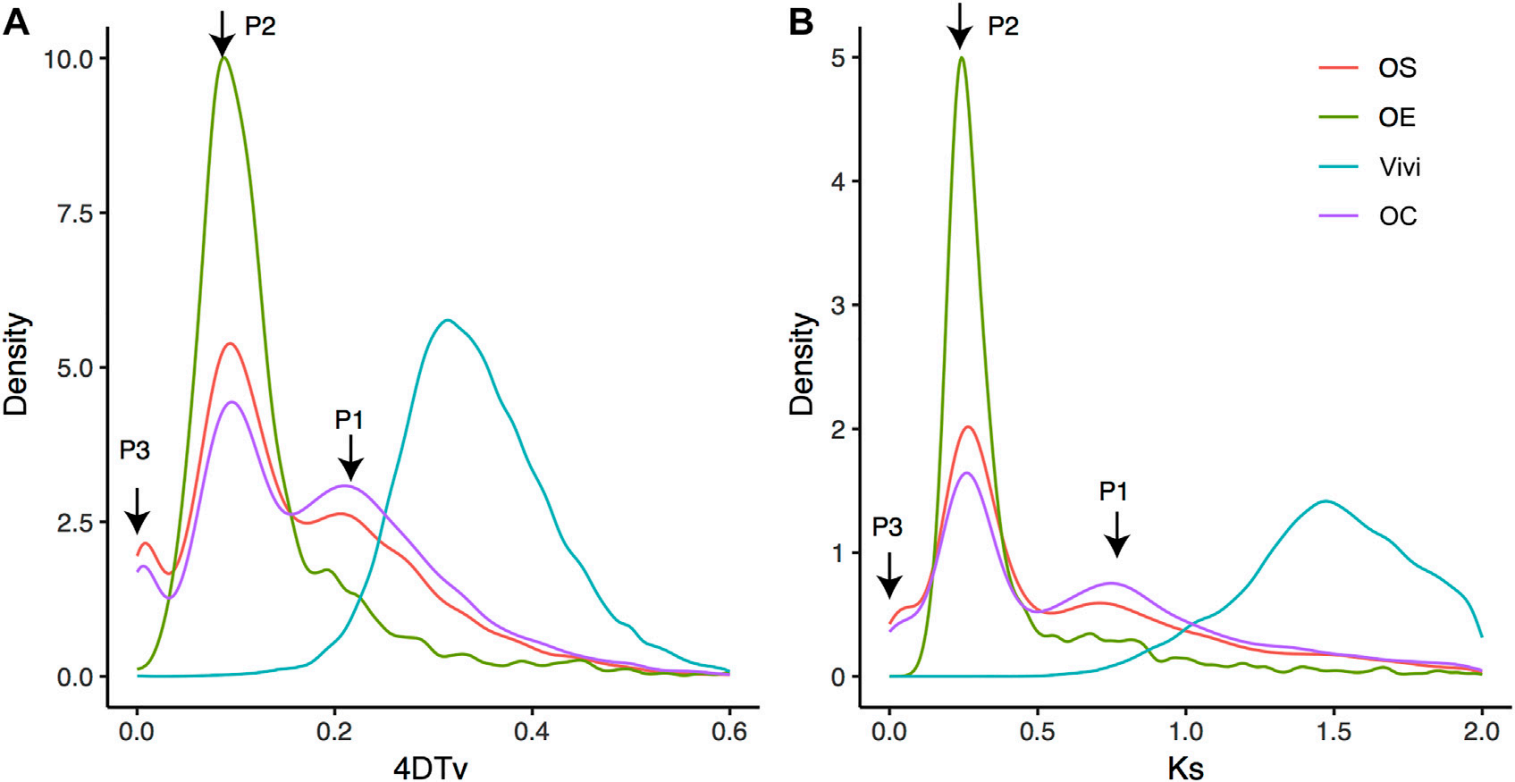
Transversion of four-fold degenerate site (i.e., 4DTv) distribution **(A)** and Ks distribution **(B)**. Abscissa and ordinate represent 4DTv or Ks distance and the percentage of homologous gene pairs, respectively. Note: Vivi represented *Vitis vinifera*, which was used to show a more ancient WGD event. P1, P2, and P3 are three recent peaks. Species evolution in phylogeny and divergence time.

It has long been known that whole-genome duplication is one of the most important evolutionary forces driving phenotypic diversity during plant speciation. Previous reports have revealed that the OS genome contains WGD events that are specific to Oleaceae (Unver et al., 2017). Here, we identified collinear blocks at the intraspecies level for three annotated genomes (OC, OS, and OE). Then, based on paralogous genes within these collinear blocks, we analyzed the whole-genome evolution events using 4DTv (transversion of four-fold degenerate site) and Ks (synonymous substitution rate) values (Figure 3). Both 4DTv and Ks demonstrated two major peaks (P1 and P2) for OC, OS, and OE, supporting their status as a species complex. In addition, OE and OC have a third minor peak (P3). No observable P3 peak in OS is possible due to synteny loss caused by the fragmented nature of the OS current reference (scaffold N50 is only 228.62 Kb). Most likely, the peaks indicate three rounds of WGD events at the same time in the genome evolution of Oleaceae species.

To examine the possibility of whole-genome triplication (WGT) underlying the three peaks, we analyzed the depth of collinear genes within the three peaks. The depths were determined with the “dissect multiple alignment” function of MCScanX based on collinear blocks of OC self-alignment (Wang et al., 2012). If WGT causes the three peaks, most of the genes of the peaks would have a collinear depth of 2, corresponding to a total of three collinear blocks. Interestingly, different from the expectation of WGT, depth 1 (1107 in P1, 2165 in P2, and 40 in P3) is higher than depth 2 (987 in P1, 888 in P2, and 15 in P3) for all the three peaks, suggesting that three rounds of WGD may have a significant role in shaping OC genome evolution. We also uncovered a dominant proportion of OC genes (73.76%, 24015 genes), retained due to the WGD events or segmental duplications, than other types of duplicates (5331 transposed duplications, 1673 tandem duplications, and 1535 proximal duplications). This composition of paralogs is similar to the pattern previously reported in *Glycine max*, which was also attributable to the WGD event (Wang et al., 2012). Absolute time inference revealed that P1, P2, and P3 occur at 69.38–81.88 Mya, 34.69–40.94 Mya, and 4.34–5.12 Mya, respectively.

To understand the phylogeny of Oleaceae (OC, OS, and OE) in eudicots, we organized a dataset A covering other five related species, namely, *Jasminum sambac*, *Forsythia suspensa*, *Fraxinus pennsylvanica*, *Fraxinus excelsior*, and *Osmanthus fragrans*, with *Arabidopsis thaliana* as an outgroup species (Supplementary Table S1B). To address the issue of unavailability of public gene annotations for these species, we used a “proxy” method. We identified 1,463 single-copy orthologous groups based on dataset B of 11 species/subspecies with their publicly available annotations (Supplementary Tables 1B, 3). Then, orthologous genes from dataset B were mapped to dataset A and inferred orthologous genes for eudicot species with OrthoFinder (Emms and Kelly, 2019).

**FIGURE 4:**
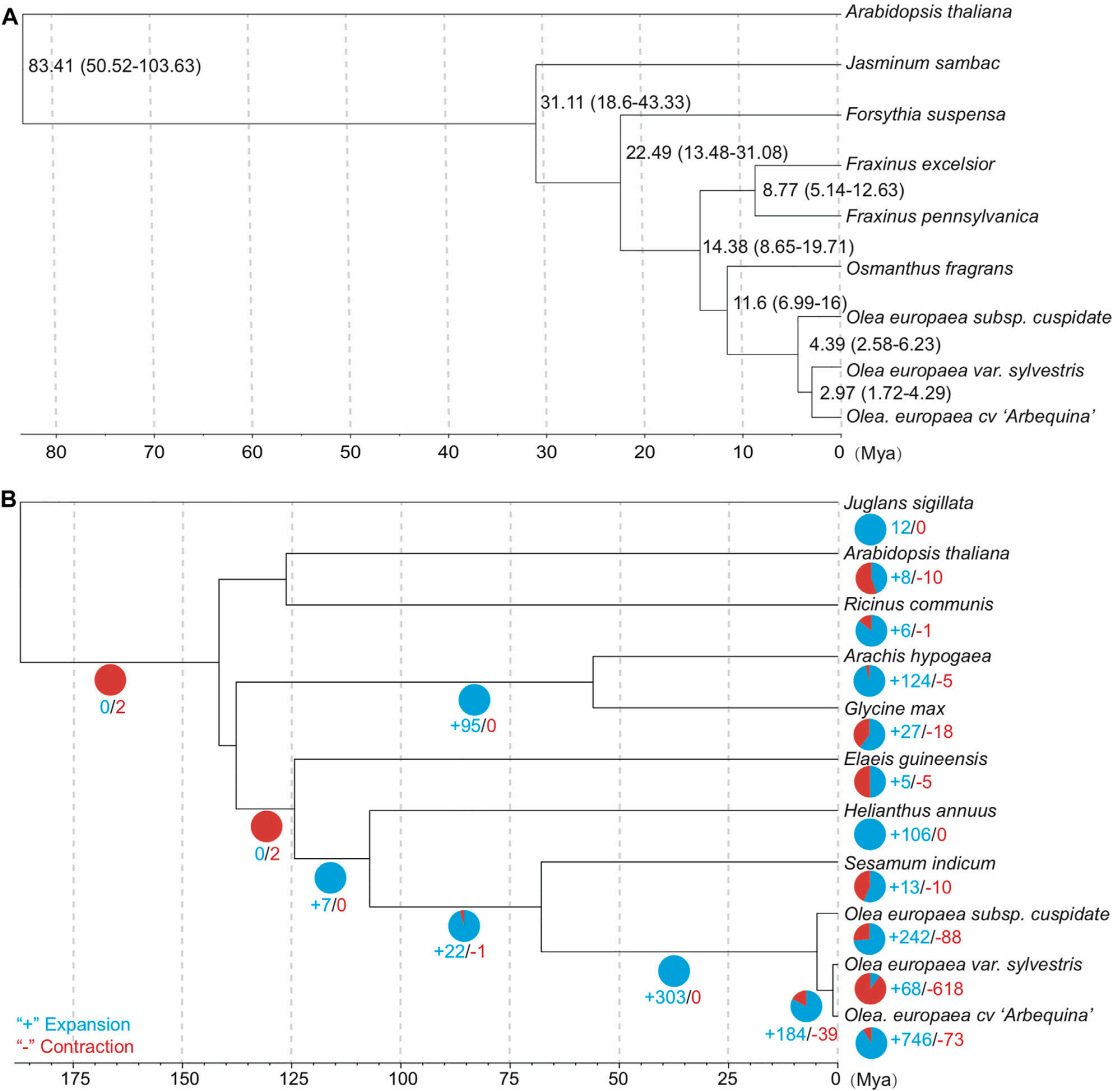
**(A)** Time-tree of OC and its related subspecies/species (dataset A). The median and 95% confidential interval (95% CI) are shown near the nodes. **(B)** Contraction and expansion statistics of gene families based on dataset B due to their available gene annotations.

Finally, we obtained 1,247 groups of “one-to-one” single-copy orthologous genes to estimate the topology and divergence time of eudicots based on dataset A (Supplementary Table S2). The phylogeny and divergence time were estimated using the approximately maximum-likelihood method for each single-copy gene group (Whelan and Goldman, 2001; Yang, 2007). The closest relationship was found among the olive subspecies, consistent with our expectation about the recent evolution of the olive complex (Figure 4A). The divergence time between OC and the last common ancestor of OE and OS was estimated to be 4.39 Mya (95% CI: 2.58–6.23 Mya). Interestingly, this time range is roughly the same with P3 peak at 4.34–5.12 Mya, suggesting the contribution of the most recent WGD event on the divergence of olive subspecies.

### Gene Family Evolution in Terms of Expansion and Contraction

**FIGURE 5:**
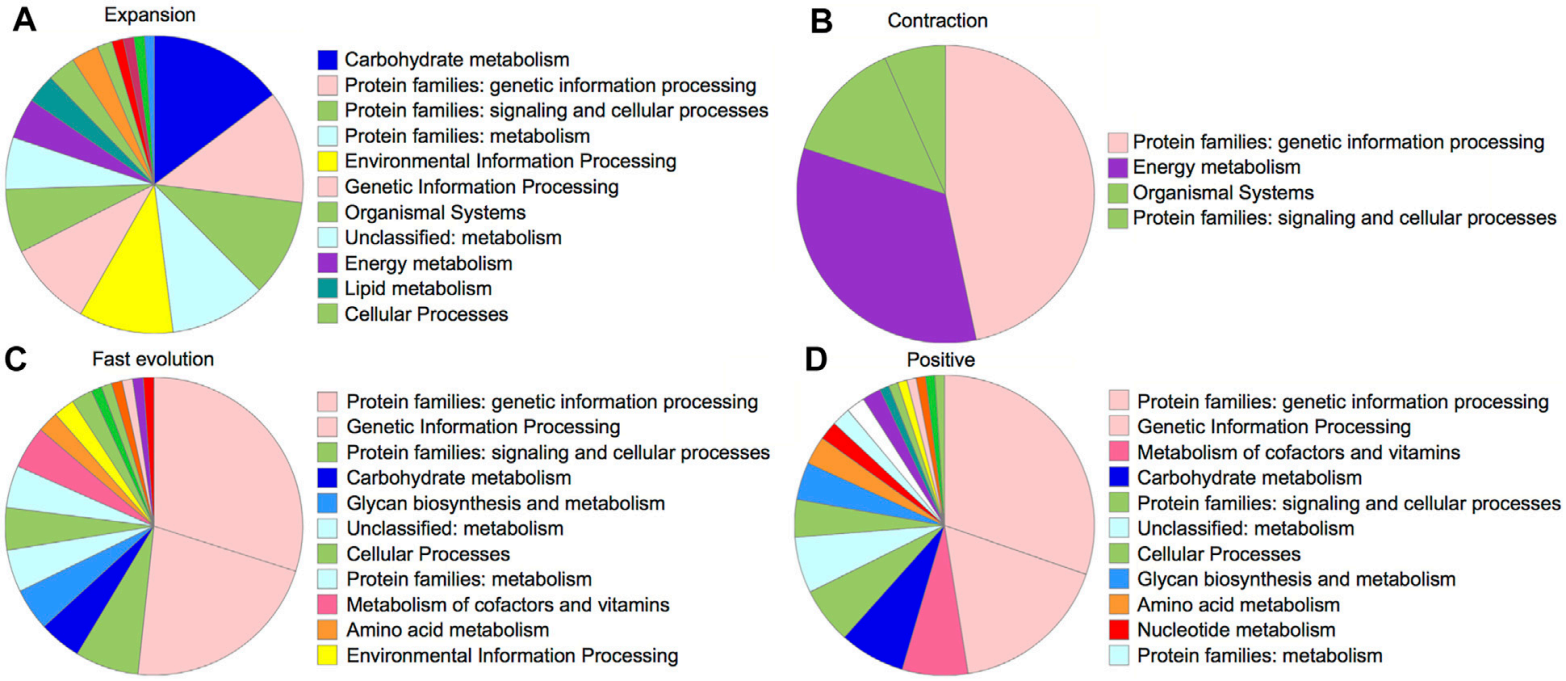
KEGG pathways for 242 gene families under expansion **(A)**, 88 gene families under contraction **(B)**, 192 genes with signals of faster evolution in OC **(C)**, and 73 positively selected genes **(D)**.

For gene family evolution, we analyzed the expansion and contraction patterns based on 11 species/subspecies of dataset B due to their available gene annotations. The ultrametric tree was estimated with r8s to transform the species tree into a time tree (Sanderson, 2003). We identified 242 gene families that expanded and 88 gene families that contracted during OC genome evolution after OC speciation (Figure 4B). For the expanded gene families, the KEGG analyses (Figure 5A) based on BlastKOALA (Kanehisa et al., 2016) revealed that the enriched pathways include carbohydrate metabolism, energy metabolism, lipid metabolism, nucleotide metabolism, amino acid metabolism, and genetic information processing. The expanded genes include alcohol dehydrogenase, isocitrate dehydrogenase (NAD+), S-(hydroxymethyl) glutathione dehydrogenase, dihydropyrimidine dehydrogenase (NADP+), polyphenol oxidase, L-ascorbate oxidase, homocysteine methyltransferase, phospholipid: diacylglycerol acyltransferase, *etc* (Supplementary Table S5). The contracted gene families majorly involve genetic information processing and energy metabolism (Figure 5B, Supplementary Table S6). These results indicate that some gene families related to traits with potential economic value, such as lipid metabolism, are under gene expansion rather than contraction, which may need further study and exploration.

### Gene Sequence Evolution Related to Selection

To identify genes under OC-specific positive selection, we conducted branch model and branch-site model tests using CODEML in PAML software (Yang, 2007). Among 1,463 “one-to-one” orthologous genes, 40.05% of genes (586) were detected to significantly deviate from the null model of neutral evolution *via* the branch model analysis by comparing the “free-ratio model” with the “one-ratio model” (*p* < 0.05, χ^2^ test). The “free-ratio model” allows the Ka/Ks ratio to be flexibly modeled, thus providing a Ka/Ks ratio for each branch to compare. By ranking Ka/Ks ratios across species, we found 13.12% of genes (192) with the highest Ka/Ks in the OC genome, suggesting their OC-specific fast evolution. 45.30% of these genes can be mapped into KEGG biological processes, including genetic information processing, glycan biosynthesis and metabolism, carbohydrate metabolism, and lipid metabolism (Supplementary Table S7, Figure 5C). The pathway analysis revealed that these genes could be categorized into 84 pathways, with metabolism and biosynthesis of secondary metabolites as the top two pathways with the most abundant genes (18 and 7 genes, respectively). Among the 192 significantly faster evolution genes (*p* < 0.05), 125 genes have Ka/Ks ratios >1, suggesting that these genes are under positive Darwinian selection.

We further conducted the branch-site model analysis by focusing only on the OC branch to identify OC-specific positively selected genes. The branch-site model detected that 7.18% of orthologous genes (105) may be under significant positive selection during OC evolution, with only 17 being shared with the branch model result, suggesting the importance of using complementary methods during the positive selection analysis. There are 73 genes, out of 105 positively selected genes identified with the branch-site model, showing at least one site with a significant positive selection signal (probability >0.95) inferred with the Bayes Empirical Bayes (BEB) analysis. KEGG analysis revealed that the pathways of these positively selected genes are related to the processes involving genetic information processing and the metabolism of cofactors and vitamins (Figure 5D). Interestingly, consistent with the expectation of oil-related traits in OC, some positively selected genes are related to lipid metabolism processes, including glycerophospholipid metabolism, ether lipid metabolism, and sphingo-lipid metabolism (Supplementary Table S7). These results indicate the potential medical and economic values of OC for further research and utilization.

**FIGURE 6:**
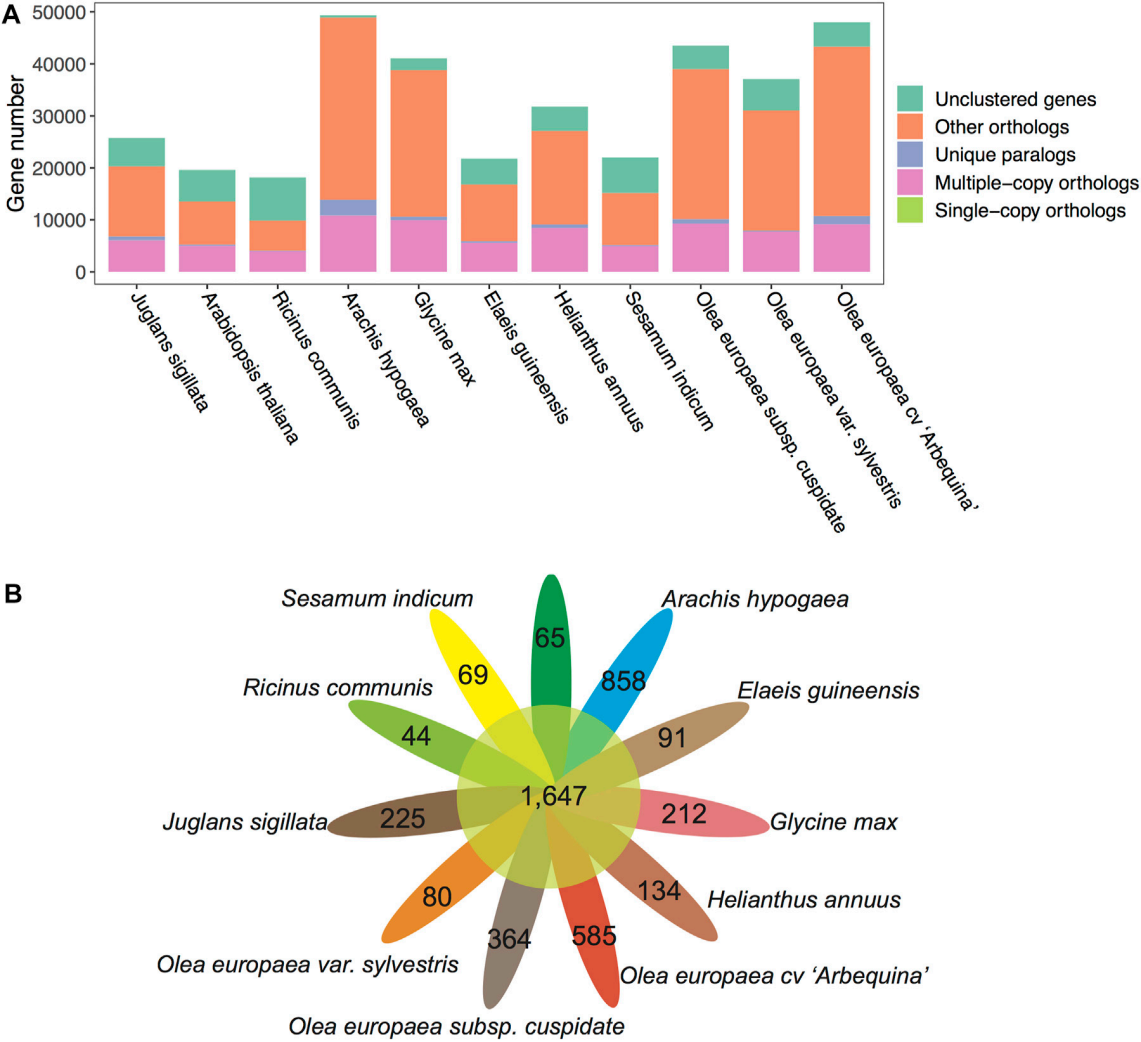
Gene family counts of OC and other plants. **(A)** Gene family compositions for 11 plant species/subspecies. **(B)** Petal diagram of gene families shared (central circle) and unique to each species/subspecies (side).

To understand the evolution of gene families, we conducted a comparative genomics analysis by incorporating other well-annotated genomes. Based on the Markov Cluster Algorithm (MCL), a fast and scalable unsupervised cluster algorithm for graphs, we identified a total of 73,994 distinct gene families (BLASTP *E*-*value*≤1e-10) (Table 4 and Figure 6A). Based on the constitution of shared or unique gene families, we found that OC has comparable numbers with OE in terms of gene family number, gene numbers within families, and unclustered gene numbers, strongly reflecting their much better gene annotation and assembly quality than OS. For a unique gene family in each species, OC is 0.62 times lower than OE (364 vs. 585) but 4.55 times higher than OS (364 vs. 80) (Figure 6B).

**TABLE 4:** Summary of gene numbers and gene family numbers.

Species	No. of families	No. of genes	No. of genes in families	Unclustered genes<	Unique family
*Arabidopsis thaliana*	3,906	19,614	13,532	6,082	65
*Arachis hypogaea*	11,047	49,359	48,914	445	858
*Elaeis guineensis*	4,741	21,783	16,793	4,990	91
*Glycine max*	9,424	41,092	38,809	2,283	212
*Helianthus annuus*	5,822	31,783	27,110	4,673	134
*Juglans sigillata*	5,612	25,769	20,310	5,459	225
*Ricinus communis*	3,051	18,161	9,847	8,314	44
*Sesamum indicum*	4,585	22,010	15,187	6,823	69
*Olea europaea subsp. cuspidate*	8,988	43,511	39,008	4,503	364
*Olea europaea* subsp. *europaea* var*. sylvestris*	7,432	37,104	31,025	6,079	80
*Olea europaea* subsp. *europae* cv ‘Arbequina'	9,386	48,032	43,344	4,688	585

### The Evolutionary Dating of Transposed Genes

**FIGURE 7:**
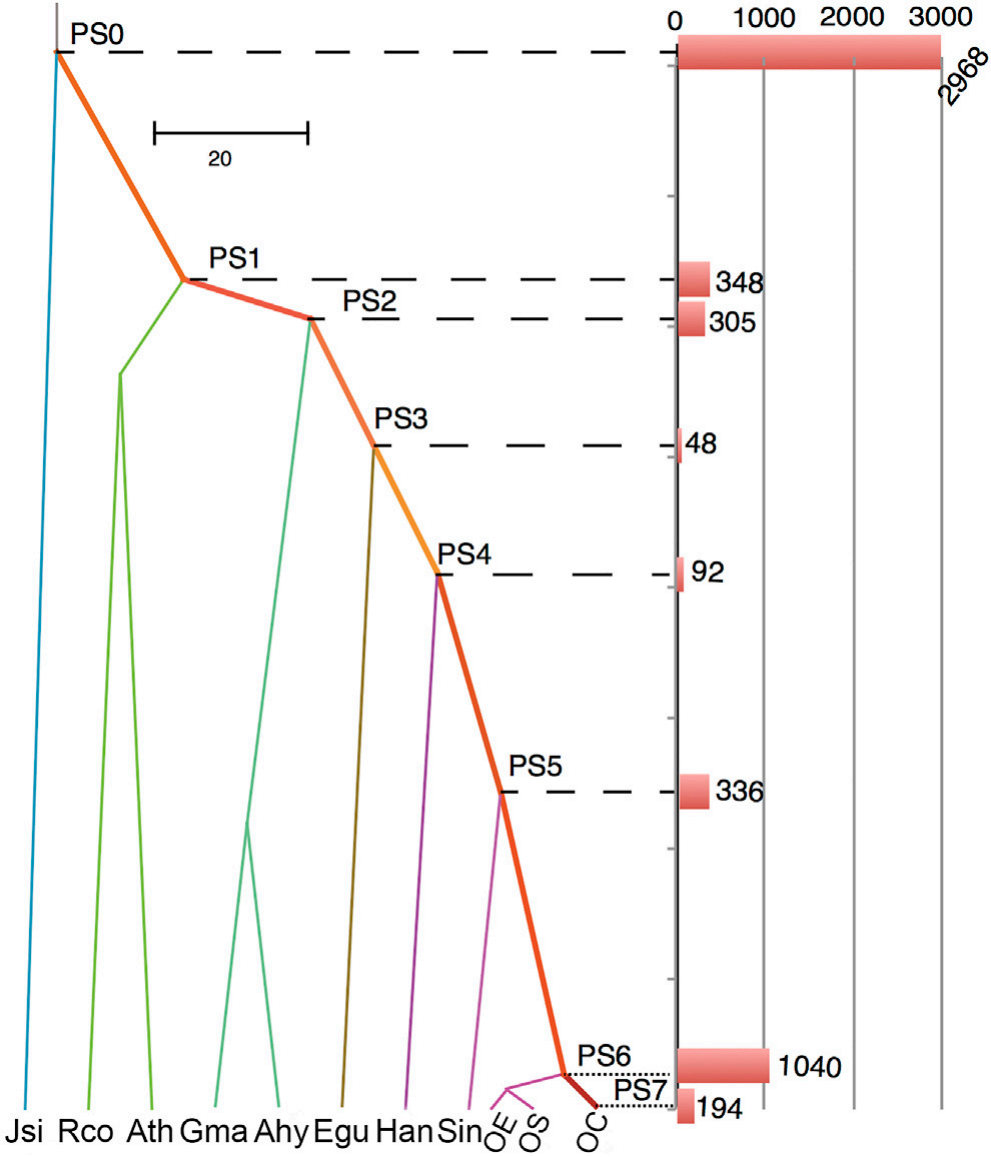
Evolutionary dating of transposed duplicated genes. PS indicates phylogenetic stages of ancestral nodes. The bars show the number of transposed genes that originated at these stages. The abbreviations and their full names are Ahy (*Arachis hypogaea*), Ath (*Arabidopsis thaliana*), Egu (*Elaeis guineensis*), Gma (*Glycine max*), Han (*Helianthus annuus*), Jsi (*Juglans sigillata*), Rco (*Ricinus communis*), and Sin (*Sesamum indicum*).

New genes, including gene duplications, are known as one of the most important drivers of phenotypic innovations in species and populations (Chen et al., 2022a; Long and Langley, 1993; Long et al., 2013; Xia et al., 2016; Chen et al., 2019; Xia et al., 2021). To understand how new gene duplications have contributed to the evolution of OC, we categorized the genes into segmental duplication, tandem duplication, proximal duplication, and transposed duplication through synteny sharing or breaking of protein-coding genes. The transposed duplicated genes were further mapped into the phylogenetic tree that leads to our focal genome OC. Hence, we can understand how OC gradually disseminated duplicated genes into new chromosome context by DNA- or RNA-based transposition processes. RNA-based transposed genes (1111 genes), which are known as retroposed genes or retrogenes (Emerson et al., 2004; Chen et al., 2019), were found to account for 20.84% of all gene transpositions. Among eight evolutionary branches leading to OC, we found a substantial outburst (19.5%) of gene transposition events in PS6 (Figure 7), which is the common ancestor of olive subspecies, suggesting the importance of new gene evolution in shaping olive speciation. Interestingly, this outburst of new genes seems to occur simultaneously with the minor WGD event (P3) that happened at 4.34–5.12 Mya. A previous study in bamboos has revealed the connections between recent WGD and new gene origination in both time and function (Jin et al., 2021). Our study provides further evidence on the close relationship between transposition and WGD events, which is worthy of further investigation.

### The Structural Variation Identification

Although the “assembly-to-assembly” approach has been successfully used to identify SVs in other species (Chen et al., 2022b; Goel et al., 2019), we failed to obtain results from this method, probably due to the known phenomenon of higher rearrangements in plants than in animals. We further identified structural variations (SVs) using Sniffles V2.0.3 (Sedlazeck et al., 2018) and a dual-alignment strategy implemented in Vulcan (Fu et al., 2021). Vulcan explores the advantages of two efficient mappers, Minimap2 (Li, 2018) and NGMLR (Sedlazeck et al., 2018), to improve the accuracy and efficiency of mapping. Here, after mapping OC long reads to OS and OE, we obtained four types of SVs, namely, deletions, duplications, insertions, and inversions (Table 5; Supplementary Tables S8, S9). We found that the number of three types of SVs (deletions, insertions, and inversions) between OC and OS is lower than that between OC and OE, suggesting a comparatively closer relationship between OC and OS. This finding is consistent with our synteny mapping result that the nucleotide identity is higher between OC and OS than between OC and OE (Figure 2A). It is well-established that SVs have higher functional impacts than SNPs (Alonge et al., 2020; Chen et al., 2022a). Thus, it is promising to identify the SVs associated with critical traits at the population level, based on larger sample size. Since reliable SV calling procedures require a high-quality genome reference, our study may pave the way for further studies of population genomics, genomic selection, and functional genomics.

**TABLE 5:** Number summary of SVs (>50bp) numbers between OC and other two references (OE and OS).

Reference	Deletions	Duplications	Insertions	Inversions
OS	41,283	67	34,866	100
OE	70,180	59	52,152	149

## Conclusion

The olive complex includes both wild and domestic subspecies, distributed in a wide range of temperate regions globally. *Olea europaea* subsp. *cuspidata* (OC) is one of the closest wild relatives of the olive tree (*O. europaea* subsp. *europaea,* OE), the symbol of peace and prosperity. Despite its close relationship with OE and great value in crossbreeding, OC still has no high-quality genomic reference, hindering its application in breeding and performance improvement. In this study, we used the most cutting-edge technologies in genomic sequencing, including Nanopore long-reads, Hi-C, second-generation sequencing, and RNA-seq, to conduct *de novo* genome assembly for an OC sample. The reference quality of OC is comparable to that of OE in terms of parameters, including scaffold N50 (50.46 Mb) and completeness of protein-coding genes (96.7%). On chromosome 17, we uncovered a particularly large region of “gene-desert” and “LTR-hotspot,” possibly associated with the two genes *in situ*, phytochrome B–like gene and arginine methyltransferase–interacting related gene, which are related to chromatin compaction and gene methylation. We uncovered the recent divergence of OC from wild and domestic olive trees at 4.39 Mya, consistent with the complicated diversification process of all olive subspecies. The reference of OC would promote its future use in both scientific research and breeding applications.

## Statement for Material Collection

Leaves of a single plant of *Olea europaea* subsp. *cuspidate* from Kunming arboretum, Yunnan Province, China (N 25°9′13″, E 102°45′9″) were collected for genome sequencing. Five kinds of tissues, namely, leaves, roots, twigs, bark, and fruits from the same plant were collected for RNA-seq to aid gene annotation. A specimen identified by Dr. Yong-Kang Sima was deposited at the Herbarium of the Yunnan Academy of Forestry and Grassland, Kunming City, China, under the voucher number Wu20056.

## Data Availability

The datasets presented in this study can be found in online repositories. The names of the repository/repositories and accession number(s) can be found in the article/Supplementary Material.
