# Supplementary figures and images for: The De Novo Genome Assembly of Olea europaea subsp. cuspidate, a Widely Distributed Olive Close Relative
Source: Front Genet. 2022 Aug 25;13:868540. doi: 10.3389/fgene.2022.868540 (PMC9454953; doi:10.3389/fgene.2022.868540)

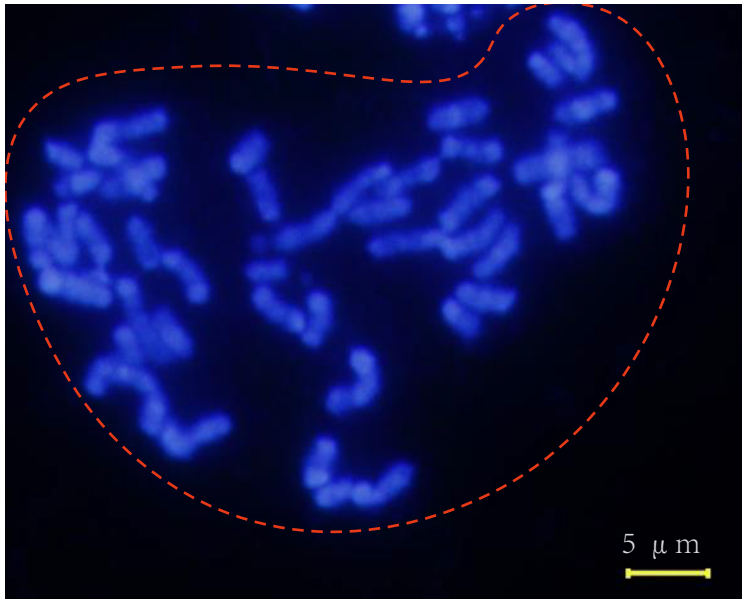

Supplement: Supplementary file 1 [file DataSheet2.pdf]
